# Quality of life assessment for colorectal cancer follow‐up: A latent profile analysis of EORTC measures

**DOI:** 10.1111/codi.70553

**Published:** 2026-07-23

**Authors:** M. H. Elise van Driel, Lissa Wullaert, Kelly R. Voigt, Pascal G. Doornebosch, Koen C. M. J. Peeters, Jennifer M. J. Schreinemakers, Maria Verseveld, Cornelis Verhoef, Dirk J. Grünhagen, Olga Husson, Martijn A. H. Oude Voshaar

**Affiliations:** ^1^ Department of Surgical Oncology Erasmus MC Cancer Institute Rotterdam The Netherlands; ^2^ Department of Surgery IJsselland Hospital Capelle aan den IJssel The Netherlands; ^3^ Department of Surgical Oncology Leiden University Medical Centre Leiden The Netherlands; ^4^ Department of Surgery Amphia Hospital Breda The Netherlands; ^5^ Department of Surgery Franciscus Hospital Rotterdam The Netherlands; ^6^ Department of Medical Oncology Netherlands Cancer Institute (NKI) Amsterdam The Netherlands; ^7^ Department of Public Health Erasmus University Medical Centre Rotterdam The Netherlands

**Keywords:** colorectal cancer, health related quality of life (HRQoL), latent profile analysis, personalised medicine

## Abstract

**Aim:**

To identify distinct health‐related quality of life (HRQoL) profiles among colorectal cancer (CRC) patients using patient‐reported outcome measures (PROMs), with the aim of informing more personalised follow‐up strategies.

**Methods:**

Baseline HRQoL data from CRC patients enrolled in the FUTURE‐primary study, a multicentre implementation study on home‐based follow‐up, were analysed. Patients completed the EORTC QLQ‐C30 and QLQ‐CR29 questionnaires. Aggregated symptom and function scales were developed using factor analysis and expert consensus. Latent profile analysis (LPA) was applied to identify subgroups based on PROM responses. Model selection was guided by the Bayesian Information Criterion (BIC), entropy, average posterior probabilities (AvePP), class sizes, clinical interpretability, and additional sensitivity and stability analyses.

**Results:**

Three HRQoL profiles were identified: low HRQoL (*n* = 62), intermediate HRQoL (*n* = 95), and high HRQoL (*n* = 42). The profiles followed a gradient‐like pattern, with consistently high, moderate, or low scores across symptom burden and functional outcomes. Classification quality was high, as indicated by high entropy (>0.93) and average posterior probabilities (>0.96), although profile assignment should be interpreted probabilistically.

**Conclusion:**

This study highlights variation in HRQoL among CRC patients. LPA identified three profiles that primarily reflected differences in overall symptom burden and functioning. These profiles may provide a preliminary framework for describing differences in supportive care needs, but their clinical applicability for tailoring follow‐up or allocating care resources requires longitudinal validation.

**Implications for Cancer Survivors:**

Identifying HRQoL profiles may support more personalised follow‐up, ensuring targeted care for those with higher symptom burden and lower functional scores, while reducing unnecessary interventions for others.

**What does this paper add to the literature?**

Colorectal cancer patients in follow‐up showed heterogeneity in patient‐reported health‐related quality of life. Latent profile analysis identified three clinically interpretable HRQoL profiles, reflecting low, intermediate and high levels of symptom burden and functioning. These profiles may help identify patients with greater supportive care needs and provide a basis for more personalised follow‐up, however longitudinal validation is needed.

## INTRODUCTION

Health‐related quality of life (HRQoL) assessments provide important insight into the physical, emotional, and social well‐being of cancer survivors. In oncology, patient‐reported outcome measures (PROMs) are increasingly used to monitor symptoms, understand patient experiences, and support clinical care. Previous studies have also shown associations between HRQoL, survival outcomes, and patient satisfaction [1, 2, 3, 4].

In colorectal cancer (CRC) care, the European Organisation for Research and Treatment of Cancer Quality of Life Questionnaire Core 30 (EORTC QLQ‐C30) and Colorectal 29 (QLQ‐CR29) are validated instruments that assess a broad range of HRQoL domains, including bowel, urinary, and sexual symptoms [5, 6]. These domains are particularly relevant during CRC follow‐up, as standard surveillance mainly focuses on recurrence detection through carcinoembryonic antigen (CEA) monitoring and symptoms such as altered bowel habit, weight loss, and fatigue [7]. HRQoL assessment may therefore provide a more comprehensive view of patients' well‐being during follow‐up.

Previous research on HRQoL in CRC has largely focused on group‐level analyses, which describe average effects across populations [8, 9]. However, systematic reviews have shown substantial heterogeneity in HRQoL outcomes among CRC patients, and it remains unclear whether meaningful HRQoL profiles can be identified within this population [10, 11]. Latent Profile Analysis (LPA) may help explore such heterogeneity by identifying patterns in patient‐reported outcomes.

In this study, we applied LPA to baseline EORTC QLQ‐C30 and QLQ‐CR29 questionnaire data from the FUTURE‐primary study [12, 13]. Our goal was to identify patient profiles based on HRQoL scores and to explore whether these profiles could provide a framework for describing variation in patient‐reported outcomes during CRC follow‐up.

## METHODS

### Patient selection

The FUTURE‐primary study is a prospective, multicentre implementation study involving CRC patients in follow‐up after surgical treatment with curative intent [13]. The study was conducted in five hospitals in the South‐West region of the Netherlands. Adult patients eligible for standard‐of‐care follow‐up were included between April 2021 and June 2024. Patients with metastatic disease were excluded.

Patients were included in the FUTURE‐primary study within 6 months after surgical resection of their primary colorectal tumour. As part of this study, the EORTC QLQ‐C30 and QLQ‐CR29 questionnaires were administered at inclusion (baseline) to assess HRQoL and monitor symptoms, providing insights into patient‐reported outcomes throughout follow‐up.

For the current analysis, only those patients who completed these questionnaires at baseline were included, as these initial responses formed the basis of the LPA aimed at identifying distinct HRQoL profiles. Missing data were imputed using predictive mean matching (PMM) with 20 iterations, generating a single imputed dataset. Single imputation was considered to be sufficient, due to the relatively low proportion of missing data (<5%). Given the limited extent of missingness, the impact of imputation uncertainty on the results was expected to be minimal, and single imputation was therefore deemed appropriate for this exploratory analysis.

### Statistical analysis

LPA was performed using symptom scales, functional scales, and aggregated scales, calculated from the responses to the EORTC‐QLQ‐C30 and EORTC‐QLQ‐CR29 questionnaires. Functional and symptom scales were calculated according to the questionnaire manuals provided by the EORTC [14]. A detailed overview of the contents of these two questionnaires is provided in Appendices S1 and S2.

To complement the functional and symptom scales, additional aggregated scales of symptom scores were developed using exploratory factor analysis (EFA) and expert consensus. EFA was used to identify underlying patterns within the symptom data, grouping related items together based on their correlations. The decision to use factor analysis was driven by the fact that many symptom scores in the EORTC questionnaires consist of only one or two items, which are typically unreliable when assessed individually [6]. To address this limitation, factor analysis allowed for the identification of clusters of symptoms that were consistently related and more robustly measured. After determining the factors, expert judgement was used to ensure that the aggregated scales reflected clinically meaningful symptom clusters. The experts, comprising oncologists and researchers, provided input on which symptoms to combine, based on clinical relevance and coherence. As these aggregated scales were developed specifically for the present analysis and are not part of the standard EORTC scoring methodology, their construct validity and reproducibility were further explored through reliability assessment and sensitivity analysis using the standard EORTC scales.

The global health scale was excluded, as it was judged to be insufficiently specific for identifying distinct patient profiles. Unlike the individual symptom and functioning scales, it reflects a broad overall appraisal of health and quality of life, which may be influenced by multiple underlying domains. This notion is supported by the Wilson & Cleary model, which views global health as an overarching concept influenced by more specific domains such as physical functioning [15]. On the original scales, low functionality scores indicate lower functioning, while high scores indicate higher functioning. For symptom scales, the opposite applies: a high score means more symptoms (a worse outcome), and a low score means fewer symptoms (a better outcome). To simplify interpretation, all symptom scales were reverse coded. As a result, lower symptom scores now indicate worse patient health, meaning more symptoms. For all scores, a median outcome with an interquartile range (IQR) was calculated.

To assess the internal consistency of the newly defined scales constructed for this study, we employed the reliability function from the semTools package in R [version 4.3.3 (2024‐02‐29)]. Although the EORTC QLQ instruments are well‐validated, the derivation of new multi‐item scales within our analyses required an independent evaluation of their internal consistency. For this purpose, McDonald's Omega was preferred over Cronbach's Alpha, as Alpha tends to underestimate the lower bound of reliability, particularly in the presence of skewed item distributions or violations of essential tau‐equivalence. Omega provides a more robust and accurate estimation under these conditions [16].

After completion of the preparatory steps, LPA was applied to the symptom scales, functional scales, and aggregated scales to identify distinct patient profiles based on the questionnaire data. The LPA was performed using the mclust package in R [version 4.3.3 (2024‐02‐29)] for model‐based clustering and classification. Standardised data based on Z‐scores were used, and model selection was based on the Bayesian Information Criterion (BIC), in combination with entropy, average posterior probabilities (AvePP), and class sizes to assess classification quality and model stability.

Additional diagnostic analyses were performed to support model selection. These included visual inspection of the candidate profile plots and posterior classification probabilities, a leave‐one‐scale‐out sensitivity analysis using the adjusted Rand index (ARI), and comparison of baseline characteristics across the candidate profile solutions.

To assess the robustness of the findings, a sensitivity analysis was performed using the standard EORTC QLQ‐C30 and QLQ‐CR29 scales without aggregation. The same LPA procedure was applied to these conventional scales.

Furthermore, baseline characteristics were analysed across the identified HRQoL profiles to examine their associations with profile membership, to gain insights into factors underlying HRQoL differences, and to determine whether patients within different profiles could be more precisely characterised. If univariable analyses showed no significant predictors, multinomial logistic regression was not pursued. Instead, a linear mixed model was applied for the continuous variable ‘Age,’ treating profile membership as a fixed effect to account for potential differences between profiles. Categorical variables were compared using Chi‐squared tests.

Lastly, the discriminative value of each symptom scale, functional scale, and aggregated scale was assessed by calculating the root mean squared error (RMSE) for all items, which measures how well each scale distinguishes between the identified HRQoL profiles. A lower RMSE indicates better differentiation between profiles, while a higher RMSE suggests less ability to distinguish between them. Pearson correlation tests were performed to evaluate the relationship between RMSE and the reliability coefficient (McDonald's Omega) for scales consisting of multiple items.

## RESULTS

The FUTURE‐primary study initially included 216 patients from five hospitals in the South‐West region of the Netherlands. Approximately 70% of patients who received information about the study consented to participate. During the study, seven patients withdrew consent, and an additional ten patients did not complete the baseline questionnaires. As a result, 199 patients were included in this analysis. The baseline characteristics of these 199 patients, who completed the EORTC QLQ‐C30 and QLQ‐CR29 questionnaires, are presented in Table 1.

**TABLE 1:** Baseline characteristics.

Total number of patients	199
Age, median (IQR)	71 (65.5–81.0)
Sex, *n* (%)	
Male	108 (54.3)
Female	91 (45.7)
Tumour stage (T), *n* (%)	
pT0	1 (0.5)
pT1	17 (8.5)
pT2	78 (39.2)
pT3	88 (44.2)
pT4a	6 (3.0)
pT4b	7 (3.5)
Lymph nodes (N), *n* (%)	
pN0	153 (76.9)
pN1	38 (19.1)
pN2	6 (3.0)
Tumour location, *n* (%)	
Right colon	87 (43.7)
Left colon	64 (32.2)
Rectum	42 (21.1)
Multiple sites	2 (1.0)
Other	4 (2.0)
Neoadjuvant RT, *n* (%)	
No	184 (92.5)
Yes	15 (7.5)
Neoadjuvant chemo, *n* (%)	
No	180 (90.5)
Yes	19 (9.5)
Adjuvant chemo, *n* (%)	
No	182 (91.5)
Yes	17 (8.5)

The median age was 71 years (IQR 55.5–87.5), with 54.3% male participants. Most patients had a history of medical conditions (75.9%), were node‐negative (76.9%), and had pT2–pT3 tumours. Tumours were mainly located in the right colon (43.7%), followed by the left colon and rectum. Most patients did not receive neoadjuvant or adjuvant therapy.

### Questionnaire outcomes

The median scores for the EORTC‐QLQ‐C30 and EORTC‐QLQ‐CR29 questionnaires are presented in Table 2. The scores are relatively high, meaning that the study population was generally well‐functioning and had relatively few symptoms.

**TABLE 2:** Median scores for the EORTC‐QLQ‐C30 and CRC‐29 scales combined.

Score/scale	Median	IQR
Functional scales		
Physical functioning	86.67	20.00
Role functioning	83.33	50.00
Emotional functioning	83.33	27.08
Cognitive functioning	83.33	33.33
Social functioning	83.33	33.33
Anxiety	66.67	33.33
Weight	100	33.33
Body image	100	22.22
Symptom scales		
Fatigue	66.67	33.33
Dyspnoea	100	33.33
Insomnia	66.67	33.33
Financial difficulties	100	0
Sexual interest	33.33	33.33
Hair loss	100	0
Bloating	100	33.33
Sexual functioning	100	33.33
Aggregated scales		
Problems with eating	91.67	16.67
Pain	91.67	20.83
Defaecation complaints	90.48	16.67
Urination complaints	88.89	16.67

### Reliability scores

Most of the functional, symptom, and aggregated scales had reliability scores above 0.70. However, three aggregated scales showed lower internal consistency: pain (*ω* = 0.61), defaecation complaints (*ω* = 0.67), and urination complaints (*ω* = 0.56). A detailed description of all reliability scores, can be found in Appendix S3.

### Latent profile analysis

**FIGURE 1:**
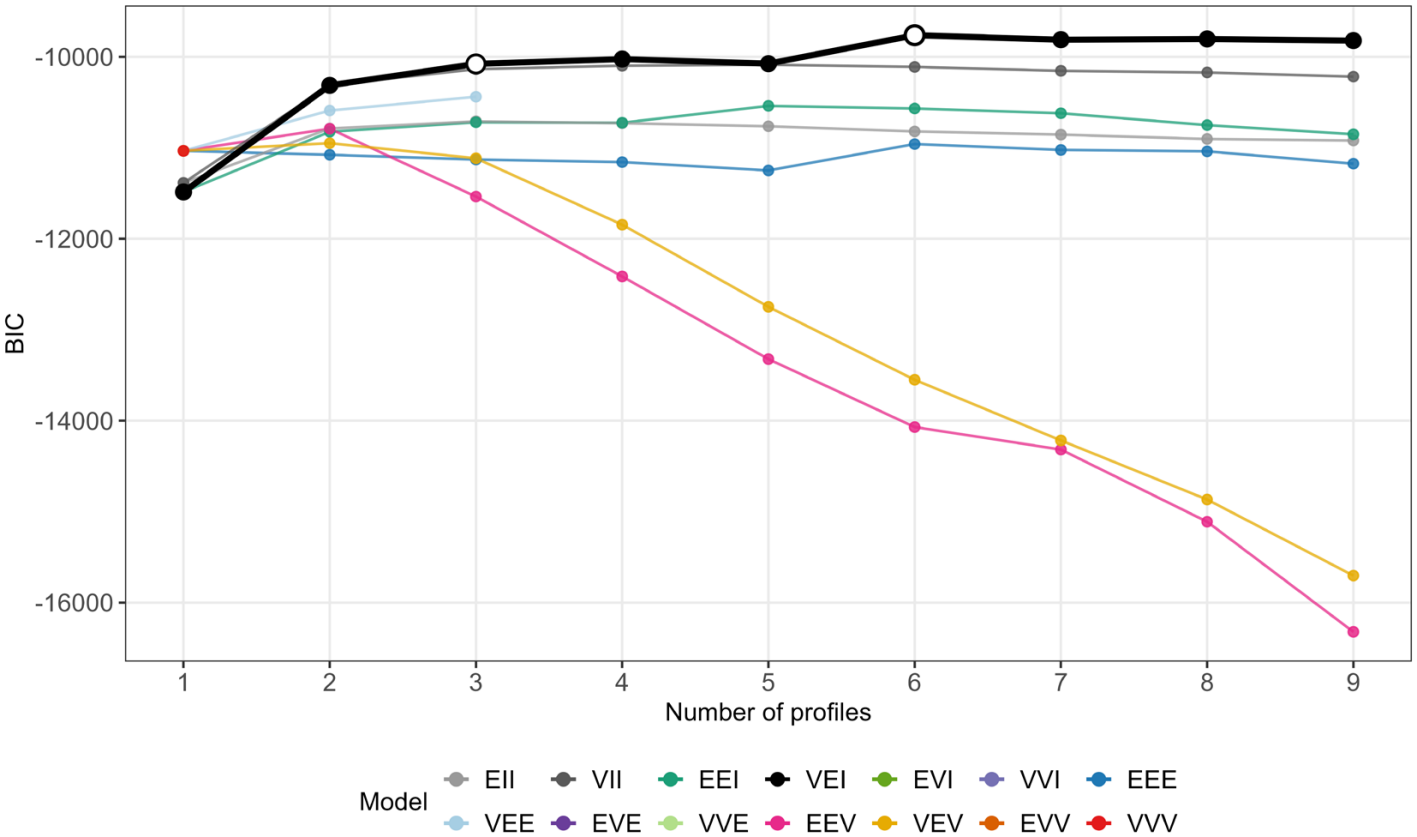
BIC plot after LPA of questionnaire outcomes. This plot shows the model fit for different numbers of profiles, with the *x*‐axis representing the number of profiles, the *y*‐axis displaying the corresponding BIC scores, and the legend indicating the various model structures (EII, VII, EEI, VEI, EVI, etc.).

LPA was performed on the questionnaire outcomes, including the aggregated scales. Among the VEI candidate models with 3–6 profiles, the highest BIC score was observed for the six‐profile model (BIC = −9762.57; Figure 1). The VEI model allows the volume of the profiles to vary while constraining their shape and orientation to be equal across profiles. All candidate VEI models demonstrated high entropy (>0.93) and high average posterior probabilities (>0.96), indicating good classification quality.

Models with more profiles resulted in smaller subgroup sizes, with the smallest group decreasing from 42 patients in the three‐profile model to 20 patients in the six‐profile model. Although the six‐profile VEI model had the highest BIC, it was not retained as the primary model. It largely followed the same low‐to‐high HRQoL gradient as the three‐profile solution, with additional distinctions mainly driven by single‐item scales (Figure S1). Classification certainty was generally high in both solutions (Figure S2), but the six‐profile model had smaller subgroups and was more sensitive to scale selection (Appendix S4).

As baseline characteristics did not reveal a clear clinical explanation for the additional profiles, the three‐profile solution was retained as the most parsimonious and clinically interpretable model based on BIC, class sizes, and overall classification quality (entropy and average posterior probabilities), and was used for all subsequent analyses (Table 3).

**TABLE 3:** Model fit and classification quality indices for candidate latent profile models.

Model	No. profiles	BIC	Entropy	Mean AvePP	No. patients per profile
VEI	3	−10078.17	0.944	0.980	62/42/95
VEI	4	−10024.97	0.932	0.970	25/71/68/35
VEI	5	−10075.15	0.942	0.968	50/26/74/27/22
VEI	6	−9762.57	0.957	0.975	37/26/20/68/26/22

*Note*: Entropy and average posterior probabilities (AvePP) reflect classification certainty, with higher values indicating better separation between profiles. Class sizes are reported to assess model stability.

**FIGURE 2:**
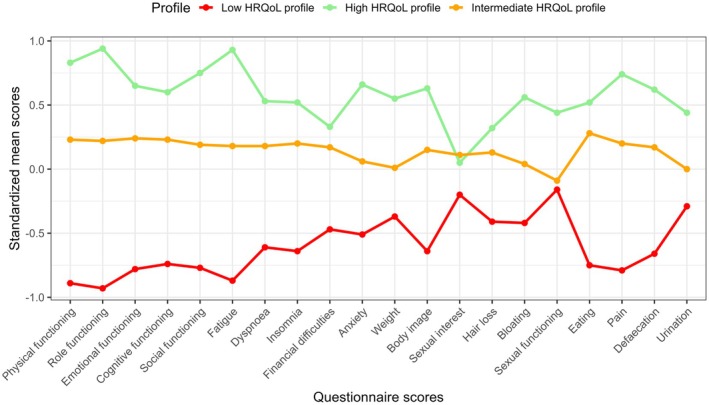
Three profiles identified through LPA and their questionnaire scores.

Figure 2 shows the three‐profile model, characterised by a gradient pattern with generally low, intermediate, and high scores across profiles. Profile 1 (*n* = 62) represented the low HRQoL group, with higher symptom burden and poorer functioning. Profile 2 (*n* = 42) represented the high HRQoL group, with fewer symptoms and better functioning. Profile 3 (*n* = 95) represented the intermediate HRQoL group, with moderate symptom burden and functional limitations. Given the probabilistic nature of LPA, these groups should be interpreted as estimated profiles rather than strictly discrete patient categories.

The sensitivity analysis using standard EORTC scales yielded a similar three‐profile structure with a comparable gradient pattern, supporting the robustness of the main findings (Figure S3).

### Profiles and baseline characteristics

Significant differences were observed for age and tumour location across the HRQoL profiles. Patients in the low HRQoL profile had a mean age of 67 years, compared to 73 years in the high HRQoL profile and 70 years in the intermediate HRQoL profile. Linear mixed model analysis showed that patients in the high HRQoL profile were older than those in the low HRQoL profile, while no significant difference was observed for the intermediate profile.

Tumour location also differed significantly between profiles (*p* = 0.020), with the low HRQoL profile showing more right‐sided tumours, the high HRQoL profile more left‐sided tumours, and the intermediate HRQoL profile more rectal tumours.

No significant differences were observed for gender, medical history, tumour stage, lymph node involvement, neoadjuvant radiotherapy, neoadjuvant chemotherapy, and adjuvant chemotherapy (Appendix S5).

### Discriminating value of questionnaire scores

Profiles primarily differed in symptom severity. Higher scale reliability was associated with better profile discrimination, as reflected by a strong correlation between RMSE and McDonald's Omega for multi‐item scales (*r* = 0.77; Appendix S6).

## DISCUSSION

This study demonstrated that a model with three distinct patient profiles‐ labelled low, intermediate, and high HRQoL‐, fit the baseline EORTC questionnaire data well. The profiles were characterised by broad differences in symptom burden and functioning across multiple domains, suggesting that they largely reflect variation in overall HRQoL severity rather than highly domain‐specific symptom patterns. These findings highlight the potential of profile‐based approaches to capture heterogeneity in patient‐reported outcomes, though their value for tailoring follow‐up care requires further longitudinal validation.

Although the six‐profile model had the highest BIC, additional diagnostics suggested that its extra profiles mainly reflected item‐driven subdivisions of the same overall HRQoL gradient rather than distinct clinical phenotypes. Combined with sensitivity to scale selection, small subgroup sizes, and no clear clinical explanation from baseline characteristics, this supported retaining the three‐profile solution. The sensitivity analysis using standard EORTC scales yielded a similar three‐profile structure, suggesting that the main findings were not solely dependent on the aggregated scales. However, as these scales were developed specifically for this study and some showed lower internal consistency, they should be considered exploratory and require validation in independent cohorts.

The results are consistent with existing literature, which suggests that younger age and right‐sided tumours are associated with a higher symptom burden and poorer outcomes [17, 18]. The identified profiles demonstrated broad differences across all scales, with no single scale showing unique discriminative power between the three profiles. This pattern suggests that the profiles are best understood as reflecting differences in overall HRQoL severity, rather than representing clearly distinct symptom phenotypes. These findings align with previous research that has documented inter‐scale correlations in the QLQ‐C30 questionnaire [19] and between the QLQ‐CR29 and QLQ‐C30 scales [20].

### Potential clinical implications

Although the use of aggregated symptom scales and exclusion of the global health scale deviates from standard EORTC methodology and should, therefore, be interpreted with caution. This approach was intended to improve specificity and clinical relevance by enhancing the robustness of symptom measurement and focusing on functional domains [15]. However, because these aggregated scales were constructed specifically for this study, their reproducibility and construct validity do require further evaluation.

**FIGURE 3:**
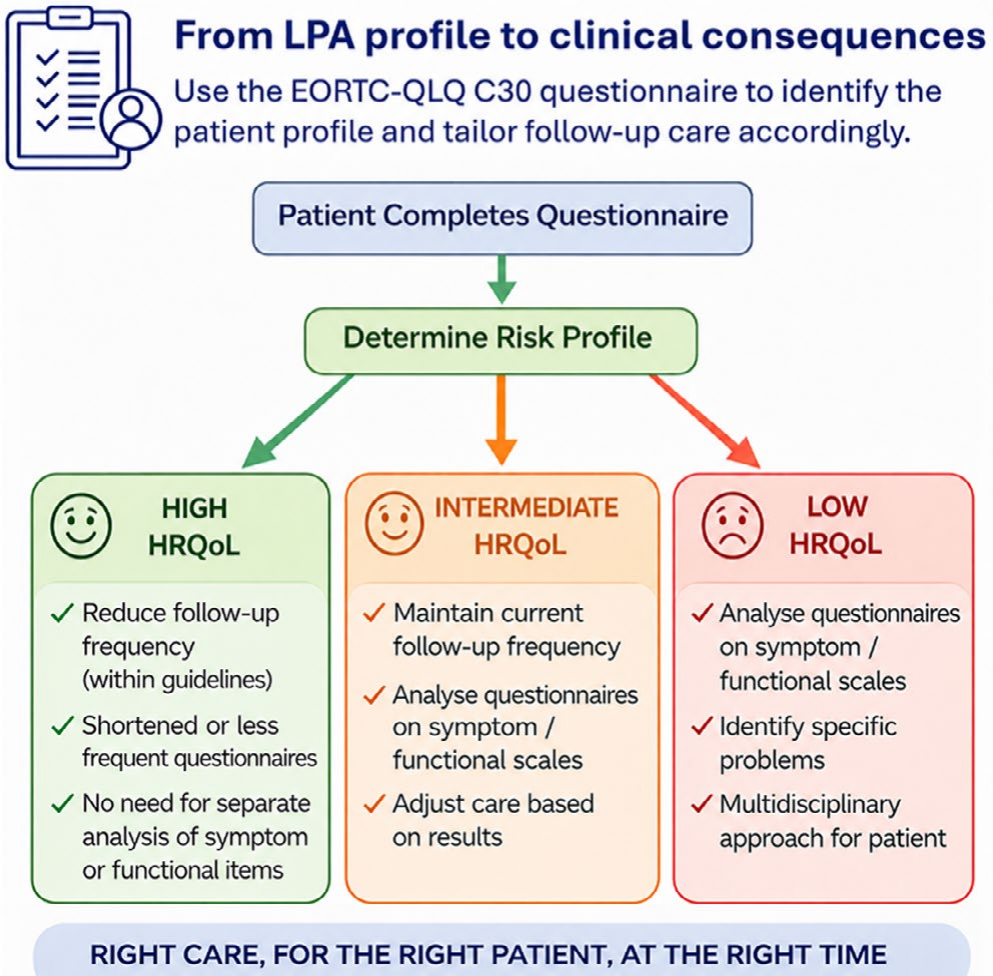
Hypothetical framework for the potential clinical use of HRQoL profiles.

The identified profiles may support more targeted care by providing a framework for stratifying patients according to overall HRQoL severity (Figure 3). Patients in the low HRQoL profile may represent a group with greater supportive care needs, whereas patients in the high HRQoL profile may have fewer current HRQoL‐related concerns. However, because this study used cross‐sectional baseline data, it cannot determine whether profile membership predicts future HRQoL deterioration, recurrence, healthcare use, or intervention needs. Therefore, the use of these profiles to guide follow‐up intensity or resource allocation should be considered exploratory and requires longitudinal validation before clinical implementation.

### Limitations

There are several limitations to consider when interpreting the results of this study. First, the relatively small group sizes within each profile may limit the generalisability of the findings, particularly for less common patient characteristics. In addition, the number of variables included in the LPA was relatively high in relation to the sample size, which may have increased the risk of overfitting and sample‐specific profile solutions. This supported the choice for the more parsimonious three‐profile model, but external validation in larger cohorts remains necessary.

Second, the use of aggregated symptom scales deviated from standard EORTC scoring methodology. Although these scales were intended to improve symptom measurement, they were study‐specific and have not been externally validated. Some, particularly urination complaints, showed relatively low internal consistency, which may have affected profile estimation. Moreover, although classification quality was high, LPA assigns patients to profiles probabilistically, meaning that profile membership should be interpreted as the most likely classification rather than as a fixed clinical category.

Third, the cohort had relatively high HRQoL scores, with most patients functioning well and experiencing few symptoms. This may have limited the diversity of symptom profiles and may reduce generalisability to patients with poorer HRQoL or higher symptom burden. The high‐HRQoL profile also consisted largely of older patients, raising the possibility of response bias, as older patients may underreport symptoms or limitations due to differing expectations of health and ageing [21, 22].

Finally, missing questionnaire data were imputed. Although missingness was limited, nonresponse may have been non‐random, for example, due to difficulty understanding items or reluctance to answer sensitive questions. More importantly, this study used baseline data only, precluding assessment of profile stability over time or predictive value for future HRQoL deterioration, recurrence, healthcare use, or supportive care needs. These findings should therefore be interpreted as exploratory and hypothesis‐generating rather than as evidence for direct implementation of profile‐based follow‐up care.

### Future research directions

Future research should assess the longitudinal stability and predictive value of these profiles, including their association with future HRQoL deterioration, healthcare use, recurrence, and supportive care needs. Validation in larger and more diverse cohorts, including patients with greater variation in symptom burden and functional status, is needed to determine the reproducibility and generalisability of the profile structure. Future studies should also evaluate whether additional factors, such as socioeconomic status, psychological comorbidities, and social support, contribute to HRQoL profile membership. Ultimately, further research is needed to determine whether profile‐based stratification can inform personalised follow‐up care in clinical practice.

## CONCLUSION

This study demonstrated that a three‐profile model distinguished patients based on overall symptom burden and functioning. The findings suggest that differences are driven by overall disease impact rather than specific symptom patterns. These profiles may support more targeted care, while future research should optimise questionnaire use to balance detail and feasibility.

## AUTHOR CONTRIBUTIONS

**Kelly R. Voigt:** Writing – review and editing; investigation; funding acquisition; data curation. **M. H. Elise van Driel:** Writing – original draft; writing – review and editing; data curation; formal analysis; investigation. **Koen C. M. J. Peeters:** Writing – review and editing; resources. **Dirk J. Grünhagen:** Writing – review and editing; funding acquisition; supervision; resources; conceptualization. **Pascal G. Doornebosch:** Writing – review and editing; resources. **Olga Husson:** Conceptualization; writing – review and editing; supervision; funding acquisition; methodology. **Cornelis Verhoef:** Resources; supervision; writing – review and editing; funding acquisition. **Jennifer M. J. Schreinemakers:** Writing – review and editing; resources. **Martijn A. H. Oude Voshaar:** Writing – review and editing; supervision; conceptualization; formal analysis; investigation; methodology. **Maria Verseveld:** Writing – review and editing; resources. **Lissa Wullaert:** Funding acquisition; writing – review and editing; data curation; investigation.

## FUNDING INFORMATION

Financial support for the FUTURE‐primary study was received from KWF Kankerbestrijding (grant number 9030).

## CONFLICT OF INTEREST STATEMENT

The authors declare no conflicts of interest.

## ETHICS STATEMENT

This study was performed in line with the principles of the Declaration of Helsinki. Ethical approval was given by the Medical Ethics Review Committee of Erasmus Medical Centre, The Netherlands (2021–0499). All patients provided written approval for study participation.

## Supporting information


**Figure S1.** Six‐profile solution of the latent profile analysis. Standardised mean scores across EORTC QLQ‐C30 and QLQ‐CR29 scales are shown for the six‐profile solution. Symptom scales were reverse‐coded prior to visualisation, such that higher scores consistently reflect better HRQoL across all functioning and symptom scales.

**Figure S2.** Posterior classification certainty for the three‐ and six‐profile VEI solutions.

**Figure S3.** Sensitivity analysis using individual EORTC questionnaire scales. Standardised mean scores across EORTC QLQ‐C30 and QLQ‐CR29 scales are shown for the low, intermediate, and high HRQoL profiles. Symptom scales were reverse‐coded prior to visualisation, such that higher scores consistently reflect better HRQoL across all functioning and symptom scales.

**Appendix S1.** Functional and symptom scales for the EORTC QLQ‐C30 questionnaire.
**Appendix S2.** Functional and symptom scales for the EORTC QLQ‐CR29 questionnaire.
**Appendix S3.** Detailed description of scales, the scores they consist of and their reliability.
**Appendix S4.** Leave‐one‐scale‐out sensitivity analysis for the three‐ and six‐profile VEI solutions.
**Appendix S5.** Univariable multinomial logistic regression analyses of baseline characteristics and HRQoL profile membership.
**Appendix S6.** RMSE and reliability for functional, symptom and aggregated scales.

## Data Availability

The data that support the findings of this study are available on request from the corresponding author. The data are not publicly available due to privacy or ethical restrictions.
