# Supplementary figures and images for: Quality of life assessment for colorectal cancer follow‐up: A latent profile analysis of EORTC measures
Source: Colorectal Dis. 2026 Jul 23;28(8):e70553. doi: 10.1111/codi.70553 (PMC13396531; doi:10.1111/codi.70553)

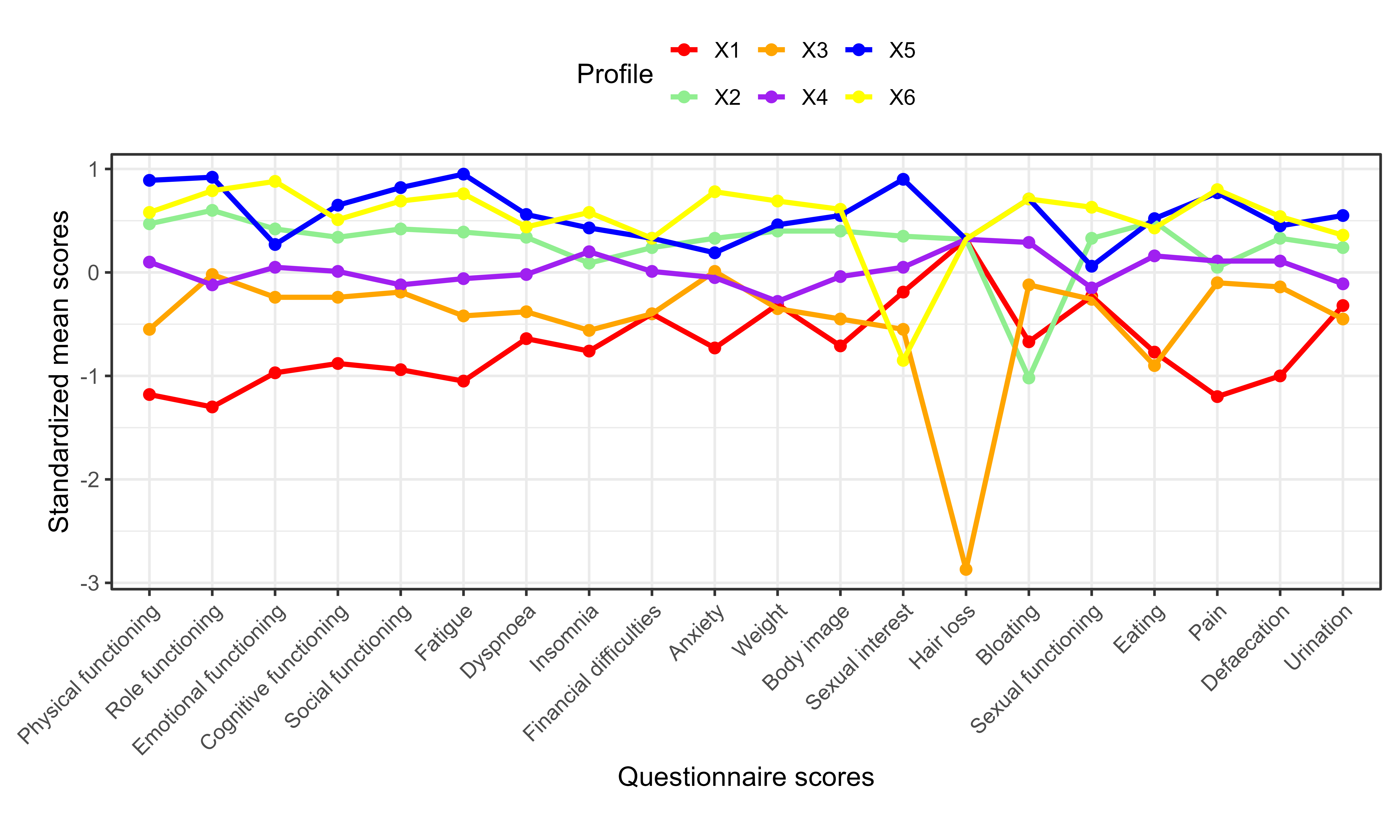

Supplement: Supplementary file 1 — Figure S1. Six‐profile solution of the latent profile analysis. Standardised mean scores across EORTC QLQ‐C30 and QLQ‐CR29 scales are shown for the six‐profile solution. Symptom scales were reverse‐coded prior to visualisation, such that higher scores consistently reflect better HRQoL across all functioning and symptom scales. [file CODI-28-0-s003.tiff]

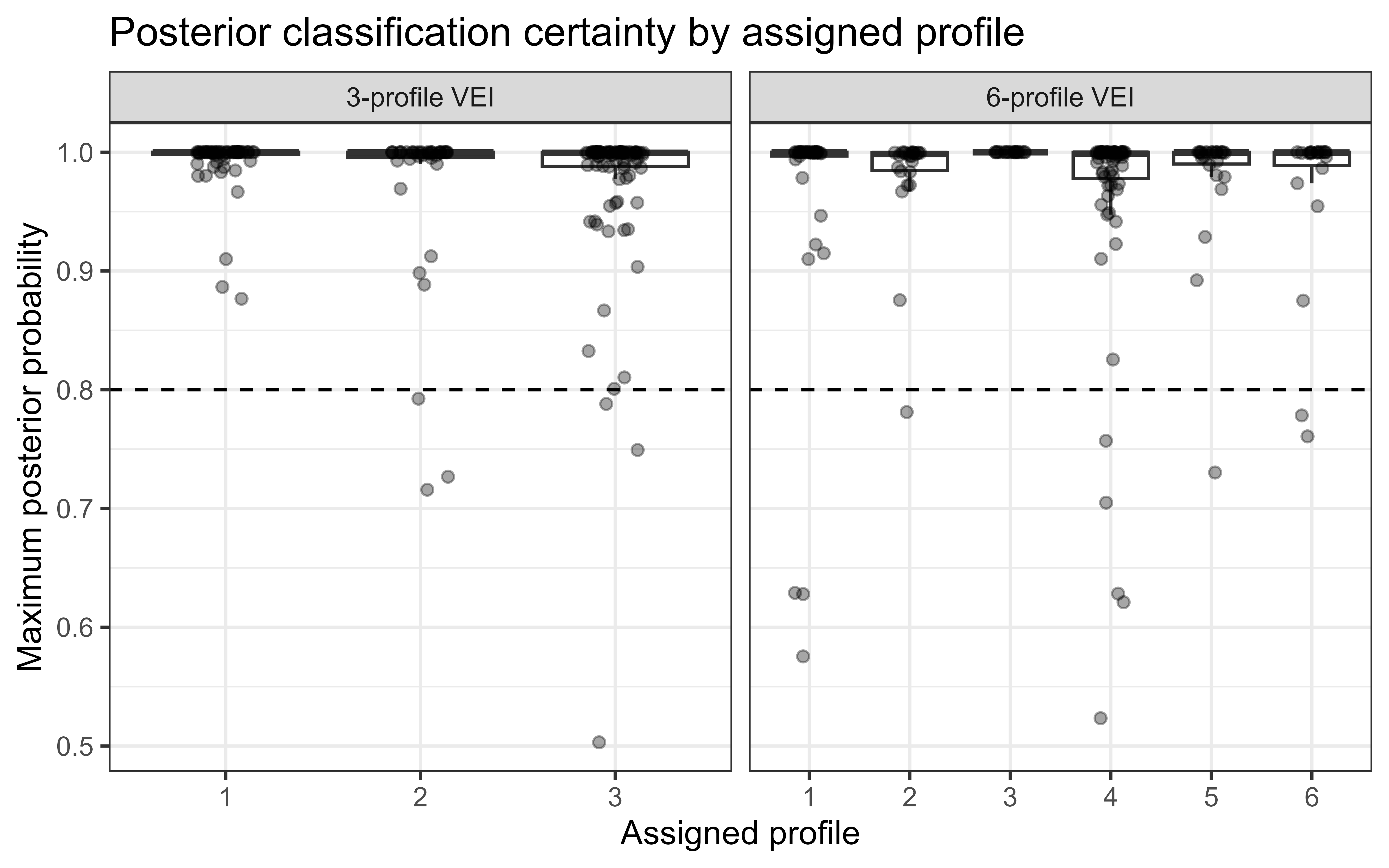

Supplement: Supplementary file 2 — Figure S2. Posterior classification certainty for the three‐ and six‐profile VEI solutions. [file CODI-28-0-s001.tiff]

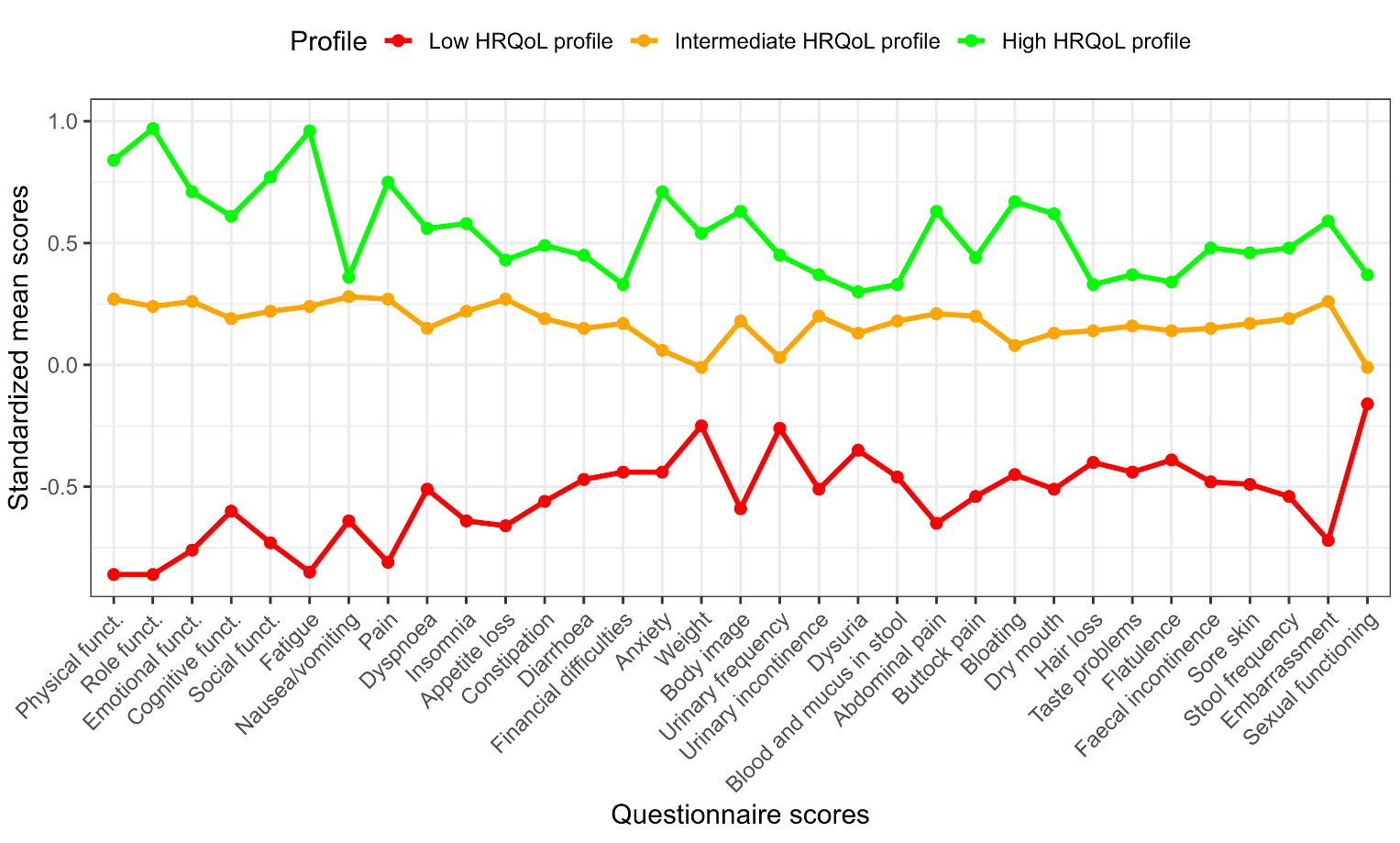

Supplement: Supplementary file 3 — Figure S3. Sensitivity analysis using individual EORTC questionnaire scales. Standardised mean scores across EORTC QLQ‐C30 and QLQ‐CR29 scales are shown for the low, intermediate, and high HRQoL profiles. Symptom scales were reverse‐coded prior to visualisation, such that higher scores consistently reflect better HRQoL across all functioning and symptom scales. [file CODI-28-0-s004.png]
