# Supplementary material for: Quality of life assessment for colorectal cancer follow‐up: A latent profile analysis of EORTC measures
Source: Colorectal Dis. 2026 Jul 23;28(8):e70553. doi: 10.1111/codi.70553 (PMC13396531; doi:10.1111/codi.70553)
Supplement: Supplementary file 4 — Appendix S1. Functional and symptom scales for the EORTC QLQ‐C30 questionnaire. Appendix S2. Functional and symptom scales for the EORTC QLQ‐CR29 questionnaire. Appendix S3. Detailed description of scales, the scores they consist of and their reliability. Appendix S4. Leave‐one‐scale‐out sensitivity analysis for the three‐ and six‐profile VEI solutions. Appendix S5. Univariable multinomial logistic regression analyses of baseline characteristics and HRQoL profile membership. Appendix S6. RMSE and reliability for functional, symptom and aggregated scales. [file CODI-28-0-s002.docx]

### **Appendix 1 Functional and symptom scales for the EORTC QLQ-C30 questionnaire**

**Functional Scales**

Global Health Status / Quality of Life (QOL) Scale:

- Q29: How would you rate your overall health during the past week
- Q30: How would you rate your overall quality of life during the past week?

Physical Functioning:

- Q1: Do you have any trouble doing strenuous activities, like carrying a heavy shopping bag or a suitcase?
- Q2: Do you have any trouble taking a long walk?
- Q3: Do you have any trouble taking a short walk outside of the house?
- Q4: Do you need to stay in bed or a chair during the day?
- Q5: Do you need help with eating, dressing, washing yourself, or using the toilet?

Role Functioning:

- Q6: Were you limited in doing either your work or other daily activities?
- Q7: Were you limited in pursuing your hobbies or other leisure-time activities?

Cognitive Functioning:

- Q20: Have you had difficulty concentrating on things, like reading a newspaper or watching television?
- Q25: Have you had difficulty remembering things?

Emotional Functioning:

- Q21: Did you feel tense?
- Q22: Did you worry?
- Q23: Did you feel irritable?
- Q24: Did you feel depressed?

Social Functioning:

- Q26: Has your physical condition or medical treatment interfered with your family life?
- Q27: Has your physical condition or medical treatment interfered with your social activities?

**Symptom Scales**

Dyspnoea (Shortness of Breath):

- Q8: Have you had shortness of breath?

Pain:

- Q9: Have you had pain?
- Q19: Did pain interfere with your daily activities?

Fatigue:

- Q10: Did you need to rest?
- Q12: Have you felt weak?
- Q18: Were you tired?

Insomnia:

- Q11: Have you had trouble sleeping?

Appetite Loss:

- Q13: Have you lacked appetite?

Nausea and Vomiting:

- Q14: Have you felt nauseated?
- Q15: Have you vomited?

Constipation:

- Q16: Have you been constipated?

Diarrhoea:

- Q17: Have you had diarrhoea?

Financial Difficulties:

- Q28: Has your physical condition or medical treatment caused you financial difficulties?

### **Appendix 2 Functional and symptom scales for the EORTC QLQ-CR29 questionnaire**

**Functional Scales**

Anxiety:

- Q43: Were you worried about your health in the future?

Weight:

- Q44: Have you worried about your weight?

Body Image:

- Q45: Have you been feeling physically less attractive as a result of your disease or treatment?
- Q46: Have you been feeling less feminine/masculine as a result of your disease or treatment?
- Q47: Have you been dissatisfied with your body?

**Symptom Scales**

Urinary Frequency:

- Q31: Did you urinate frequently during the day?
- Q32: Did you urinate frequently during the night?

Urinary Incontinence:

- Q33: Have you had any unintentional release of urine?

Dysuria:

- Q34: Did you have any pain when you urinated

Abdominal Pain:

- Q35: Did you have abdominal pain?

Buttock Pain:

- Q36: Did you have pain in your buttocks/anal area/rectum?

Bloating:

- Q37: Did you have bloating in your abdomen?

Blood and Mucus in Stool:

- Q38: Have you had blood in your stools?
- Q39: Have you had mucus in your stools?

Dry Mouth:

- Q40: Did you have a dry mouth?

Hair Loss:

- Q41: Have you lost hair as a result of your treatment?

Taste Problems:

- Q42: Have you had problems with your sense of taste?

Flatulence:

*For patients with a colostomy:*

- Q49: Have you had unintentional release of gas/flatulence from your stoma bag?

*For patients without a colostomy:*

- Q49: Have you had unintentional release of gas/flatulence from your back passage?

Faecal Incontinence:

*For patients with a colostomy:*

- Q50: Have you had leakage of stools from your stoma bag?

*For patients without a colostomy:*

- Q50: Have you had leakage of stools from your back passage?

Sore skin:

*For patients with a colostomy:*

- Q51: Have you had sore skin around your stoma?

*For patients without a colostomy:*

- Q51: Have you had sore skin around your anal area?

Stool frequency:

*For patients with a colostomy:*

- Q52: Did frequent bag changes occur during the day?
- Q53 Did frequent bag changes occur during the night?

*For patients without a colostomy:*

- Q52: Did frequent bowel movements occur during the day?
- Q53 Did frequent bowel movements occur during the night?

Embarrassment:

*For patients with a colostomy:*

- Q54: Did you feel embarrassed because of your stoma?

*For patients without a colostomy:*

- Q54: Did you feel embarrassed because of your bowel movements?

Sexual interest:

*For men:*

- Q56: to what extend were you interested in sex?

*For women:*

- Q58: Have you been sexually active?

Sexual Functioning:

*For men:*

- Q57: Have you been able to have and maintain an erection?

*For women:*

- Q56: Have you had pain during sexual intercourse?

**Appendix 3 Detailed description of scales, the scores they consist of and their reliability**

| Aggregated scales | Related scores (and questions) | Coefficient omega |
| --- | --- | --- |
|  | Physical functioning (Q1-Q5) | 0.8412 |
|  | Role functioning (Q6, Q7) | 0.7893 |
|  | Emotional functioning (Q21-Q24) | 0.8116 |
|  | Cognitive functioning (Q20, Q25) | 0.6992 |
|  | Social functioning (Q26, Q27) | 0.7619 |
|  | Fatigue (Q10, Q12, Q18) | 0.8840 |
|  | Body image (Q45-Q47) | 0.7146 |
| Problems with eating | Nausea and vomiting (Q14, Q15) | 0.7291 |
|  | Appetite loss (Q13) |  |
|  | Dry mouth (Q40) |  |
|  | Taste problems (Q42) |  |
| Pain | Pain (Q9, Q19) | 0.6133 |
|  | Buttock pain (Q36) |  |
|  | Abdominal pain (Q35) |  |
|  | Sore skin (Q51) |  |
| Defecation complaints | Constipation (Q16) | 0.6728 |
|  | Diarrhoea (Q17) |  |
|  | Blood and mucus in stool (Q38, Q39) |  |
|  | Flatulence (Q49) |  |
|  | Faecal incontinence (Q50) |  |
|  | Stool frequency (Q52, Q53) |  |
|  | Embarrassment (Q54) |  |
| Urination complaints | Urinary frequency (Q31, Q32) | 0.5625 |
|  | Urinary incontinence (Q33) |  |
|  | Dysuria (Q34) |  |

**Appendix 4 Leave-one-scale-out sensitivity analysis for the three- and six-profile VEI solutions**

| **Removed scale** | **ARI 3-profile solution** | **ARI 6-profile solution** | **Minimum class size 3-profile** | **Minimum class size 6-profile** |
| --- | --- | --- | --- | --- |
| Physical functioning | 0.876 | 0.415 | 42 | 15 |
| Role functioning | 0.268 | 0.556 | 37 | 7 |
| Emotional functioning | 0.808 | 0.581 | 41 | 15 |
| Cognitive functioning | 0.873 | 0.918 | 44 | 19 |
| Social functioning | 0.875 | 0.576 | 40 | 20 |
| Fatigue | 0.277 | 0.602 | 38 | 16 |
| Dyspnoea | 0.951 | 0.474 | 43 | 14 |
| Insomnia | 0.926 | 0.759 | 38 | 15 |
| Financial difficulties | 0.957 | 0.805 | 44 | 20 |
| Anxiety | 0.871 | 0.566 | 42 | 13 |
| Weight | 0.911 | 0.814 | 39 | 20 |
| Body image | 0.892 | 0.746 | 38 | 20 |
| Sexual interest | 0.911 | 0.468 | 41 | 13 |
| Bloating | 0.925 | 0.367 | 44 | 13 |
| Hair loss | 0.933 | 0.505 | 42 | 21 |
| Sexual functioning | 0.897 | 0.337 | 38 | 12 |
| Problems with eating | 0.890 | 0.499 | 39 | 11 |
| Pain | 0.820 | 0.806 | 36 | 15 |
| Defaecation complaints | 0.884 | 0.568 | 43 | 15 |
| Urination complaints | 0.922 | 0.580 | 41 | 20 |

Each row shows the results after removing one questionnaire scale and refitting the three- and six-profile VEI models. Agreement with the original solution was assessed using the adjusted Rand index (ARI), with higher values indicating greater similarity to the original profile assignment. Minimum class size is shown to assess the emergence of small subgroups. All models converged.**Appendix 5 Univariable multinomial logistic regression analyses of baseline characteristics and HRQoL profile membership**

| **Variable** | **Profile*** | **OR (95% CI)** | **p-value** |
| --- | --- | --- | --- |
| Age | High HRQoL | 1.06 (1.02–1.10) | 0.004 |
|  | Intermediate HRQoL | 1.03 (0.99–1.06) | 0.107 |
| Gender | High HRQoL | 0.80 (0.36–1.75) | 0.575 |
|  | Intermediate HRQoL | 0.59 (0.31–1.12) | 0.105 |
| Medical history | High HRQoL | 0.87 (0.36–2.09) | 0.755 |
|  | Intermediate HRQoL | 1.30 (0.61–2.77) | 0.489 |
| Tumour stage | High HRQoL | 1.01 (0.70–1.43) | 0.978 |
|  | Intermediate HRQoL | 0.94 (0.70–1.27) | 0.704 |
| Lymph node involvement | High HRQoL | 1.01 (0.73–1.38) | 0.970 |
|  | Intermediate HRQoL | 0.82 (0.56–1.20) | 0.303 |
| Tumour location | High HRQoL | 0.53 (0.33–0.85) | 0.009 |
|  | Intermediate HRQoL | 0.73 (0.52–1.02) | 0.067 |
| Neoadjuvant radiotherapy | High HRQoL | 0.23 (0.03–1.96) | 0.178 |
|  | Intermediate HRQoL | 0.86 (0.28–2.61) | 0.787 |
| Adjuvant chemotherapy | High HRQoL | 1.26 (0.36–4.43) | 0.717 |
|  | Intermediate HRQoL | 0.86 (0.28–2.61) | 0.787 |
| Neoadjuvant chemotherapy | High HRQoL | 0.39 (0.08–1.99) | 0.259 |
|  | Intermediate HRQoL | 0.72 (0.25–2.10) | 0.551 |

***** All profiles were compared to the reference profile: low HRQoL

**Appendix 6 RMSE and reliability for functional, symptom and aggregated scales**

| **Score / scale** | **RMSE** | **Coefficient omega** |
| --- | --- | --- |
| Physical functioning | 1.1472 | 0.8412 |
| Role functioning | 1.2474 | 0.7893 |
| Emotional functioning | 0.9545 | 0.8116 |
| Cognitive functioning | 0.8958 | 0.6992 |
| Social functioning | 1.0167 | 0.7619 |
| Fatigue | 1.2012 | 0.8840 |
| Dyspnoea | 0.7614 | * |
| Insomnia | 0.7709 | * |
| Financial difficulties | 0.5317 | * |
| Anxiety | 0.7814 | * |
| Weight | 0.6120 | * |
| Body image | 0.8480 | 0.7146 |
| Sexual interest | 0.2048 | * |
| Bloating | 0.6488 | * |
| Hair loss | 0.4852 | * |
| Sexual functioning | 0.4003 | * |
| Problems with eating | 0.8426 | 0.7291 |
| Pain | 1.0230 | 0.6133 |
| Defaecation complaints | 0.8528 | 0.6728 |
| Urination complaints | 0.4860 | 0.5626 |

* for scores consisting of only 1 question. no reliability score could be calculated
